# Concordance between ICD-10-AM clinical coding and SARS-CoV-2 PCR testing for COVID-19 in Australian hospitals

**DOI:** 10.1177/18333583251370486

**Published:** 2025-09-12

**Authors:** Getiye Dejenu Kibret, Judith Thomas, Jeffrey J Post, Kate Curtis, William Rawlinson, Andrew Georgiou, Mirela Prgomet

**Affiliations:** 1 Macquarie University, Australia; 2 Prince of Wales Hospital, Australia; 3 UNSW Sydney, Australia; 4 University of Sydney, Australia; 5 NSW Health Pathology, Australia

**Keywords:** international classification of diseases, ICD-10, COVID-19, SARS-CoV-2, pathology, patients, health information management

## Abstract

**Background:**

The introduction by the World Health Organization of specific International Statistical Classification of Diseases and Related Health Problems, 10th Revision (ICD-10) clinical codes for coronavirus disease 2019 (COVID-19) in early 2020 was key to standardising disease reporting and supporting global public health efforts. However, the concordance between these clinical codes and laboratory-confirmed COVID-19 cases based on Severe Acute Respiratory Syndrome Coronavirus 2 (SARS-CoV-2) polymerase chain reaction (PCR) results remains largely unexamined in Australia.

**Objective:**

This study evaluated the concordance between ICD-10 Australian Modification (ICD-10-AM) code U07.1 (COVID-19, virus identified) and SARS-CoV-2 PCR test results in admitted patient records, to improve case identification.

**Method:**

This retrospective study analysed routinely collected electronic medical record data from 13 public hospitals in New South Wales, Australia. Clinical coding of ICD-10-AM U07.1 was assessed using SARS-CoV-2 PCR results as the reference standard. Sensitivity, specificity, positive predictive value and negative predictive value were calculated. A mixed-effects logistic regression model was used to assess diagnostic concordance, adjusting for patient demographics.

**Results:**

Among 25,724 admissions with a SARS-CoV-2 PCR test, 39.4% were confirmed COVID-19 cases based on positive SARS-CoV-2 PCR test results. The ICD-10-AM clinical coding of U07.1 demonstrated excellent accuracy, with a sensitivity of 91.5% (95% CI: 90.8–92.2%) and 94.1% (95% CI: 93.6–94.6%) compared to conventional and rapid PCR-confirmed cases, respectively.

**Conclusion:**

The ICD-10-AM code U07.1 aligns well with SARS-CoV-2 PCR-confirmed cases, supporting its use as a reliable marker for COVID-19 in hospital data for surveillance and research purposes.

**Implications for health information management practice:**

Ongoing improvements in clinical coding practices are necessary to minimise misclassification and enhance accuracy for public health planning.

## Introduction

The introduction of specific International Statistical Classification of Diseases and Related Health Problems, 10th Revision (ICD-10) clinical codes for Severe Acute Respiratory Syndrome Coronavirus 2 (SARS-CoV-2) infection by the World Health Organization (WHO) in early 2020 (Table S1) was a critical step in standardising disease reporting and supporting global public health responses during the pandemic (World Health Organization, 2020). The codes, including U07.1 and U07.2, were adapted from pre-existing emergency clinical codes in ICD-10 and assigned specifically to coronavirus disease 2019 (COVID-19) diagnoses at that time. U07.1 was designated for laboratory-confirmed cases (i.e. SARS-CoV-2 polymerase chain reaction (PCR)-positive), while U07.2 was introduced for cases with a clinical or epidemiological diagnosis of COVID-19 in the absence of laboratory confirmation (Australian Institute of Health and Welfare (AIHW), 2022; World Health Organization, 2020). These COVID-19 ICD-10 clinical codes have facilitated data comparability across regions and health systems, providing clearer insights into the global pandemic. The continual use and adaptation of such classification systems are vital in the ongoing management of COVID-19 and other emerging infectious diseases.

Evaluating the reliability and effectiveness of clinical coding in real-world applications is essential for assessing impact on public health surveillance and response strategies. ICD-10 coded administrative health records are widely used in epidemiological research, particularly for estimating disease incidence and prevalence, monitoring drug safety, tracking long-term cohorts, and informing policy decisions. However, the utility of such data depends heavily on the accuracy of clinical coding. Inaccurate or inconsistent clinical coding can introduce bias, undermine surveillance efforts and lead to misinformed clinical and policy decisions. While the analytical performance of many SARS-CoV-2 PCR assays approaches 100% sensitivity at viral loads of 500–5000 copies/mL, their clinical performance is influenced by biological and pre-analytical factors, with sensitivity of approximately 80%, and specificity ranging between 98% and 99% (College of American Pathologists, 2025).

Several international studies have evaluated the accuracy of ICD-10 clinical code U07.1 against PCR-confirmed cases, reporting a wide range of sensitivity (approximately 49–98%) and consistently high specificity (generally ⩾92%; Bhatt et al., 2021; Kadri et al., 2020; Lynch et al., 2021; Moura et al., 2023; Regan et al., 2022). These variations highlight the influence of factors such as clinical coding practices, patient demographics and clinical contexts on the accuracy of ICD-10 clinical coding (Bhatt et al., 2021; Moura et al., 2024). A summary of selected published findings is provided in the Supplemental files (Table S2).

Australia and New Zealand use a modified version of the ICD-10, known as ICD-10 Australian Modification (ICD-10-AM). Despite the widespread use of ICD-10 coded administrative data for COVID-19 research and surveillance in Australia and New Zealand, there is limited evidence evaluating the diagnostic accuracy of the ICD-10-AM in this context. This study evaluated the concordance between ICD-10-AM clinical coding of confirmed COVID-19 cases and PCR test results in hospital admission records.

## Method

### Study design and setting

This multicentre retrospective study utilised real-world electronic medical record data from 13 public (government-funded) hospitals in New South Wales, Australia. Routinely collected data from January 2022 to July 2024 were extracted using the Admitted Patient Data Collection (APDC) and Laboratory Information System (LIS) databases. Demographic characteristics, including sex, age and inpatient facility, along with ICD-10-AM clinical codes, were obtained from the APDC (Table S1, Online Supplemental), while the SARS-CoV-2 PCR test results were sourced from the LIS.

### Population and outcome measures

The study included admitted patients at the study hospitals between January 2022 and July 2024. All SARS-CoV-2 PCR tests conducted during the hospital admission period with recorded results were included. The primary outcome–COVID-19 clinical coding concordance was determined by comparing documentation of the ICD-10-AM clinical code U07.1 to SARS-CoV-2 PCR test results, including both rapid and conventional PCR. Rapid PCR refers to a nucleic acid amplification test with a faster turnaround time than conventional PCR but remains distinct from rapid antigen tests (RATs), which are not PCR-based. An admission was considered eligible for the ICD-10-AM clinical code U07.1 if the patient tested positive for SARS-CoV-2 by PCR at any point during their hospital stay. In cases with discordant PCR results, the presence of any positive test was taken as evidence of infection. PCR results were then compared against the assigned ICD-10-AM clinical codes to assess clinical coding concordance. Demographic characteristics, including sex (male, female), age, socioeconomic grouping (low, middle, high), and year of admission (2022–2024), were incorporated into the statistical model to account for potential confounding effects. Socioeconomic grouping was determined using the Index of Relative Socio-Economic Disadvantage, derived from Socio-Economic Indexes for Areas data, defined by the Australian Bureau of Statistics (2018). Clinical coding was evaluated using:

- *Sensitivity:* The proportion of PCR-confirmed SARS-CoV-2 positive cases (from LIS) that were correctly assigned the ICD-10-AM clinical code U07.1 in the APDC database.
- *Specificity:* The proportion of PCR-confirmed SARS-CoV-2 negative cases (from LIS) that were not assigned U07.1 in the APDC database.
- *Positive predictive value (PPV):* The proportion of U07.1 coded episodes in the APDC database that were confirmed by a positive SARS-CoV-2 result in LIS.
- *Negative predictive value (NPV):* The proportion of hospital episodes where U07.1 clinical code was not assigned in the APDC database that were confirmed as SARS-CoV-2 negative by a PCR result in LIS.

### Statistical analyses

Descriptive statistics, including the mean with standard deviation (SD), the median with interquartile range (IQR; first and third quartiles) and frequencies with percentages were calculated to summarise baseline characteristics. Mixed-effects logistic regression models were fitted with SARS-CoV-2 PCR-confirmed diagnosis as the outcome variable and the ICD-10-AM U07.1 clinical code as the main explanatory variable, along with patient demographics and clinical characteristics. Random effects (patients nested within hospitals) were considered to account for variations across hospitals and between individual patients. The overall discriminatory ability of the model was assessed using the Area Under the Receiver Operating Characteristic curve (AUROC).

### Ethical considerations

Ethics approval for the study was obtained from the NSW Population and Health Services Research Ethics Committee 2022/ETH02091 substudy 2024UMB0901.

## Results

The analysis included 22,719 patients with 25,724 hospital admissions. Of these admissions, 39.4% (*n* = 10,124) had laboratory-confirmed COVID-19, as determined by positive SARS-CoV-2 PCR test results. In total, 48,082 SARS-CoV-2 PCR tests were performed during the study period, of which 33,372 (69.4%) returned positive results. The mean number of tests per patient was 2.1, while the median (IQR) was 1 (1–2). The median age of patients was 71 years (IQR: 47–83), and 11,441 (50.4%) patients were female. Most admissions (79.6%) occurred in 2022 (Table 1). Of the 13 hospitals included in the study, 83.2% of admissions were in principal referral hospitals (large hospitals providing specialised services). Over three-quarters (77%) of these hospitals, accounting for 90.8% of total admissions, were in major cities, while the remainder were situated in inner regional areas.

**Table 1.** Distribution of SARS-CoV-2 PCR tests by demographic characteristics, NSW, Australia, January 2022–July 2024.

Variable	All patients (*n* = 2,2719)	All SARS-CoV-2 PCR tests (*n* = 48,082)	Positive SARS-CoV-2 PCR tests (*n* = 33,372)	Negative SARS-CoV-2 PCR test (*n* = 14,710)
Sex, *n* (%)				
Male	11,275 (49.6)	24,795 (51.6)	16,926 (50.7)	7869 (53.5)
Female	11,441 (50.4)	23,287 (48.4)	16,446 (49.3)	6841 (46.5)
Age, median (Q1, Q3)	71 (47, 83)	75 (57, 84)	73 (54, 83)	77 (63, 85)
Socioeconomic grouping, *n* (%)				
Low	235 (1)	458 (1)	332 (1)	126 (0.9)
Mid	2982 (13.1)	6579 (13.7)	4517 (13.5)	2062 (14)
High	19,502 (85.8)	41,045 (85.4)	28,523 (85.5)	12,522 (85.1)
Year of admission, *n* (%)				
2022	18,077 (79.6)	39,393 (81.9)	30715 (92)	8678 (59)
2023	3302 (14.5)	6483 (13.5)	2060 (6.2)	4423 (30.1)
2024	1337 (5.9)	2196 (4.6)	587 (1.8)	1609 (10.9)
Facility, *n* (%)				
Hospital 1	681 (3)	1180 (2.5)	968 (2.9)	212 (1.4)
Hospital 2	3782 (16.6)	8228 (17.1)	5383 (16.1)	2845 (19.3)
Hospital 3	6123 (27)	12,567 (26.1)	8960 (26.8)	3607 (24.5)
Hospital 4	3412 (15)	6016 (12.5)	4036 (12.1)	1980 (13.5)
Hospital 5	591 (2.6)	755 (1.6)	616 (1.8)	139 (0.9)
Hospital 6	144 (0.6)	726 (1.5)	554 (1.7)	172 (1.2)
Hospital 7	116 (0.5)	584 (1.2)	448 (1.3)	136 (0.9)
Hospital 8	238 (1)	658 (1.4)	458 (1.4)	200 (1.4)
Hospital 9	89 (0.4)	476 (1)	330 (1)	146 (1)
Hospital 10	1745 (7.7)	3271 (6.8)	2188 (6.6)	1083 (7.4)
Hospital 11	4561 (20.1)	10,132 (21.1)	6931 (20.8)	3201 (21.8)
Hospital 12	1170 (5.1)	3117 (6.5)	2209 (6.6)	908 (6.2)
Hospital 13	67 (0.3)	372 (0.8)	291 (0.9)	81 (0.6)

### Concordance of ICD-10-AM clinical coding for COVID-19 with SARS-CoV-2 PCR test results

Among the 25,724 hospital admissions included in the analysis, 58.6% (*n* = 15,062) were classified as true negatives and 31.8% (*n* = 8192) as true positives, indicating substantial concordance between clinical coding and PCR testing. False positives (PCR−/ICD-10-AM+) accounted for 7.5% (*n* = 1932), while false negatives (PCR+/ICD-10-AM−) represented only 2.1% (*n* = 538) of the total admissions (Table 2). Among the 538 false negative cases, 427 (79.4%) were coded as U07.2 (COVID-19, virus not identified), 102 (18.9%) as U06.0, which is used when COVID-19 testing is conducted and results conclusively rule out infection, and 9 (1.7%) as Z03.81, which denotes suspected exposure later ruled out. These findings indicate that all false negative records were assigned alternative ICD-10-AM clinical codes, rather than the appropriate U07.1, representing potential instances of miscoding.

**Table 2.** Percentage agreement between ICD-10-AM clinical code U07.1 and SARS-CoV-2 PCR test results, stratified by demographic characteristics, NSW, Australia, January 2022–July 2024.

Variable	All admissions, *n* = 25,724	SARS-CoV-2 PCR-/ICD-10-AM- (true negative), *n* = 15,062	SARS-CoV-2 PCR+/ICD-10-AM- (false negative), *n* = 538	SARS-CoV-2 PCR-/ICD-10-AM+ (false positive), *n* = 1932	SARS-CoV-2 PCR+/ICD-10-AM+ (true positive), *n* = 8192
Sex, *n* (%)					
Male	12,880 (50.1)	7329 (48.7)	265 (49.3)	1029 (53.3)	4257 (52)
Female	12,844 (49.9)	7733 (51.3)	273 (50.7)	903 (46.7)	3935 (48)
Age, median (Q1, Q3)	72 (49, 83)	66 (39, 81)	75 (51, 84)	78 (66, 86)	77 (62, 85)
Socioeconomic grouping, *n* (%)					
Low	246 (1)	159 (1.1)	5 (0.9)	24 (1.2)	58 (0.7)
Mid	3389 (13.2)	1818 (12.1)	55 (10.2)	345 (17.9)	1171 (14.3)
High	22,089 (85.9)	13,085 (86.9)	478 (88.8)	1563 (80.9)	6963 (85)
Year of admission, *n* (%)					
2022	20,529 (79.8)	14,869 (98.7)	337 (62.6)	1103 (57.1)	4220 (51.5)
2023	3687 (14.3)	134 (0.9)	131 (24.3)	616 (31.9)	2806 (34.3)
2024	1504 (5.8)	55 (0.4)	70 (13)	213 (11)	1166 (14.2)

ICD10-AM negative: ICD-10-AM U07.1 not assigned.

Two mixed-effects logistic regression models were fitted to assess concordance between ICD-10-AM clinical code U07.1 and SARS-CoV-2 PCR test results, separately for conventional and rapid PCR tests. Both models adjusted for relevant patient-level characteristics and accounted for clustering at the hospital and individual levels using random intercepts. Fixed-effect estimates, including adjusted odds ratios and 95% confidence intervals for all candidate variables, are presented in Supplemental files (Table S3). The intra-class correlation coefficients at the hospital and patient levels were derived from the random effects and are reported in the Supplemental files (Table S4). Both models demonstrated excellent discriminative ability, with AUC values of 98.8% (rapid PCR) and 98.9% (conventional PCR) (Figure S1, Online Supplemental).

**Figure 1.**
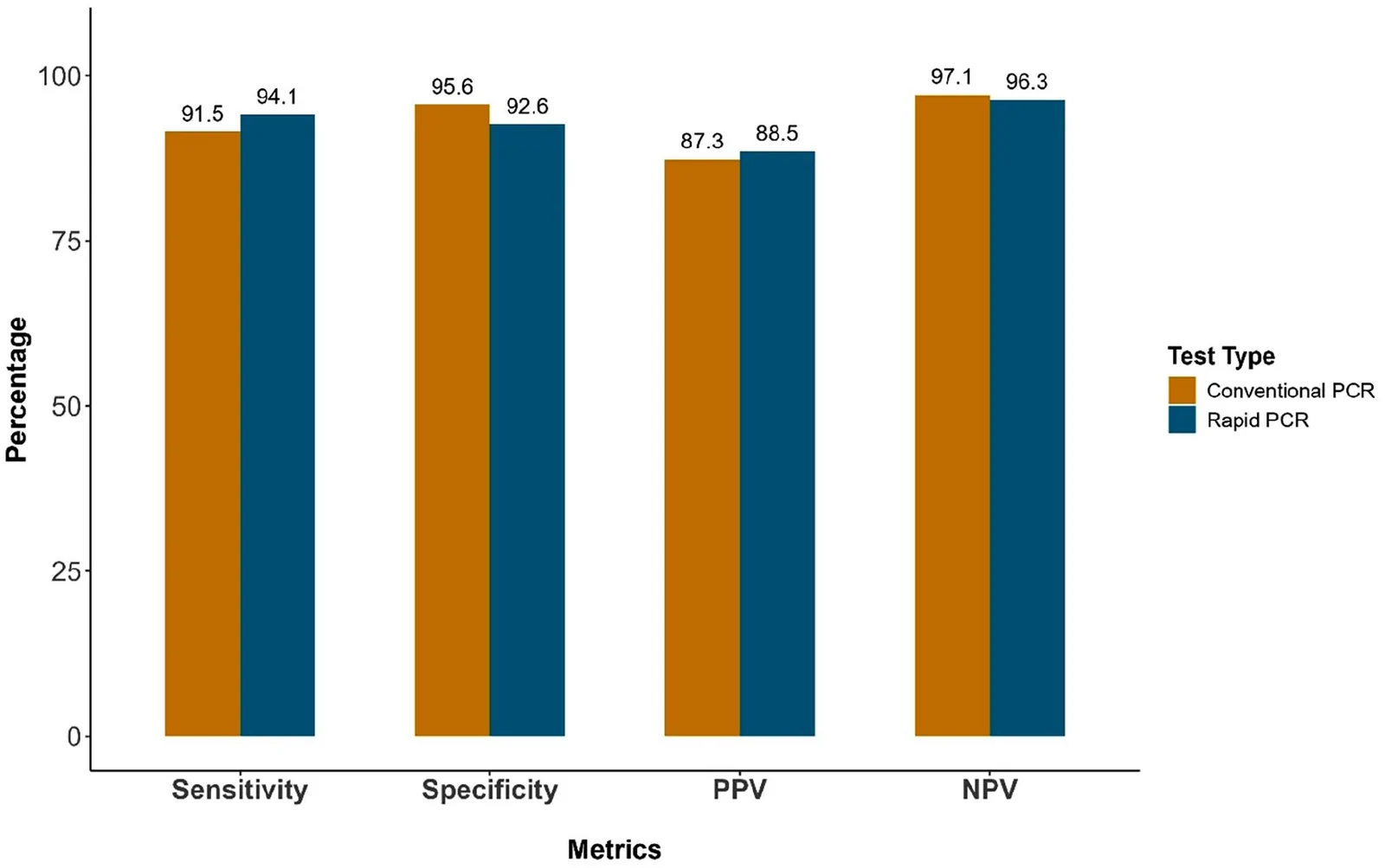
Sensitivity, specificity, positive predictive value, and negative predictive value of ICD-10-AM U07.1 clinical coding compared with SARS-CoV-2 results from conventional and rapid PCR testing. ICD-10-AM negative: ICD-10-AM U07.1 not assigned; PCR: polymerase chain reaction; SARS-CoV-2: severe acute respiratory syndrome Coronavirus 2.

The ICD-10-AM U07.1 clinical coding exhibited high sensitivity, correctly identifying 91.5% (95% CI: 90.8–92.2%) of COVID-19 cases confirmed by conventional PCR and 94.1% (95% CI: 93.6–94.6%) by rapid PCR. Specificity was also high, at 95.6% (95% CI: 95.3–95.9%) against conventional PCR and 92.6% (95% CI: 92.2–93.1%) against rapid PCR. PPV was 87.3% (95% CI: 86.5–88.1%) for conventional PCR and 88.5% (95% CI: 87.8–89.1%) for rapid PCR, reflecting the proportion of ICD-10-AM coded cases that were true positives. NPV was 97.1% (95% CI: 96.9–97.4%) for conventional PCR and 96.3% (95% CI: 96.0–96.6%) for rapid PCR, confirming a high likelihood that non-coded cases were true negatives (Figure 1).

## Discussion

We evaluated the concordance between ICD-10-AM U07.1 clinical coding for COVID-19 infections and SARS-CoV-2 PCR results, including both rapid and conventional PCR testing methods as the reference standard. Our findings indicate that ICD-10-AM clinical coding of U07.1 demonstrated high positive and negative predictive accuracy, supporting its suitability for clinical and epidemiological use.

These results support the utility of administrative data based on ICD-10-AM for large-scale COVID-19 epidemiological surveillance and retrospective research studies. However, our results have shown relatively lower accuracy compared with findings from previous studies examining COVID-19 clinical coding from other countries, including the United States and Canada, that reported sensitivity as high as 99.5% and specificity ranging between 92.8% and 99.5% (Bhatt et al., 2021; Bosch et al., 2022; Moura et al., 2023, Moura et al., 2024; Wu et al., 2022). In contrast, there was a significantly lower sensitivity value (49.2%) reported from a US study (Bhatt et al., 2021). It is interesting to note the higher sensitivity with the rapid PCR test compared to the conventional PCR test, which may relate to shorter turnaround times and clinical documentation of COVID-19 before discharge. This variability necessitates careful contextual consideration when relying on administrative data for research.

Of the 1932 cases classified as false positives, some may reflect prior community-confirmed infections not captured in the LIS but nonetheless used to inform clinical diagnosis and clinical coding. Others may have been clinically diagnosed and assigned U07.1 instead of the more appropriate U07.2. These factors likely contributed to clinical coding misclassification. False negatives – COVID-19 cases confirmed by PCR but not assigned the ICD-10-AM clinical code U07.1 – represented a relatively small proportion of the total cohort, contributing to the high NPV observed. This finding is consistent with the NPVs reported in previous studies (Bhatt et al., 2021; Moura et al., 2023, 2024).

A high NPV is important in the context of COVID-19 clinical coding, as it reflects the ability of clinical coding systems to accurately identify individuals without the disease. Accurate clinical coding is essential for surveillance, health system planning, research and reimbursement. This is especially important when evaluating the impact of COVID-19 on health outcomes, such as its effects on pregnancy outcomes or long-term complications. With the increasing usage of RATs in the diagnosis of COVID-19 it will be important to assess the clinical coding accuracy of U07.2 for COVID-19 cases diagnosed with a RAT, to provide a more complete picture of the disease epidemiology (Kendall et al., 2021; Van Honacker et al., 2021).

### Limitations of the study

The main limitation of this study is its reliance on retrospective data, which may be subject to incomplete documentation. Additionally, the data were sourced from a single Australian state and therefore may not be generalisable to other jurisdictions with different clinical coding practices. The over-representation of urban and higher socioeconomic areas may limit the applicability of our findings to more disadvantaged or rural populations. Our model included an inter-year comparison to adjust for potential differences across years. However, as COVID-19 case numbers and testing decrease, the probability of accurately detecting and categorising cases (a priori likelihood) also decreases. Thus, it will be important to ensure ongoing clinical coding accuracy. Furthermore, our study focused exclusively on cases coded with ICD-10-AM U07.1, which represents laboratory-confirmed SARS-CoV-2 infection. It does not include cases coded as U07.2, which represent clinically diagnosed COVID-19 without laboratory confirmation. Therefore, the findings reflect the accuracy of U07.1 clinical coding only and do not capture the broader spectrum of COVID-19 cases identified solely through clinical assessment.

## Conclusion

Accurate ICD-10-AM clinical coding is vital for effective public health surveillance. It strengthens the credibility of epidemiological research and drives informed clinical and policy decisions. The high concordance between the ICD-10-AM clinical code U07.1 and SARS-CoV-2 PCR- confirmed COVID-19 cases establishes its reliability for retrospective identification of COVID-19 cases in inpatient hospital settings. Nonetheless, continued improvements in clinical coding accuracy and standardisation remain essential to maximising effectiveness in epidemiological surveillance and healthcare research.

## Supplemental Material

sj-docx-1-him-10.1177_18333583251370486 – Supplemental material for Concordance between ICD-10-AM clinical coding and SARS-CoV-2 PCR testing for COVID-19 in Australian hospitals Supplemental material, sj-docx-1-him-10.1177_18333583251370486 for Concordance between ICD-10-AM clinical coding and SARS-CoV-2 PCR testing for COVID-19 in Australian hospitals by Getiye Dejenu Kibret, Judith Thomas, Jeffrey J Post, Kate Curtis, William Rawlinson, Andrew Georgiou and Mirela Prgomet in Health Information Management Journal

## References

[bibr1-18333583251370486] Australian Bureau of Statistics (2018) Socio-Economic Indexes for Areas (SEIFA) 2016, Technical paper. Australian Bureau of Statistics, Canberra, Australia.

[bibr2-18333583251370486] Australian Institute of Health and Welfare (AIHW) (2022) Australian Burden of Disease Study: Definition and coding of COVID-19 deaths, Canberra, Australia.

[bibr3-18333583251370486] Bhatt AS McElrath EE Claggett BL , et al. (2021) Accuracy of ICD-10 diagnostic codes to identify COVID-19 among hospitalized patients. Journal of General Internal Medicine 36(8): 2532–2535. 34100236 10.1007/s11606-021-06936-w PMC8183587

[bibr4-18333583251370486] Bosch NA Law AC Peterson D , et al. (2022) Validation of the international classification of diseases code for COVID-19 among critically ill patients. Annals of the American Thoracic Society 19(5): 861–863. 34851812 10.1513/AnnalsATS.202110-1147RL PMC9116339

[bibr5-18333583251370486] College of American Pathologists (2025) How good are COVID-19 (SARS-CoV-2) diagnostic PCR tests? Available at: https://www.cap.org/member-resources/articles/how-good-are-covid-19-sars-cov-2-diagnostic-pcr-tests (accessed 19 June 2025).

[bibr6-18333583251370486] Kadri SS Gundrum J Warner S , et al. (2020) Uptake and accuracy of the diagnosis code for COVID-19 among US hospitalizations. Journal of the American Medical Association 324(24): 2553–2554. 33351033 10.1001/jama.2020.20323 PMC7756233

[bibr7-18333583251370486] Kendall EA Arinaminpathy N Sacks JA , et al. (2021) Antigen-based rapid diagnostic testing or alternatives for diagnosis of symptomatic COVID-19: A simulation-based net benefit analysis. Epidemiology 32(6): 811–819. 34292212 10.1097/EDE.0000000000001400 PMC8478097

[bibr8-18333583251370486] Lynch KE Viernes B Gatsby E , et al. (2021) Positive predictive value of COVID-19 ICD-10 diagnosis codes across calendar time and clinical setting. Clinical Epidemiology 13: 1011–1018. 34737645 10.2147/CLEP.S335621 PMC8558427

[bibr9-18333583251370486] Moura CS Morrison LJ Hohl CM , et al. (2024) Administrative data ICD-10 diagnostic codes identifies most lab-confirmed SARS-CoV-2 admissions but misses many discharged from the Emergency Department. Scientific Reports 14(1): 6008. 38472258 10.1038/s41598-023-49501-7 PMC10933440

[bibr10-18333583251370486] Moura CS Neville A Liao F , et al. (2023) Validity of hospital diagnostic codes to identify SARS-CoV-2 infections in reference to polymerase chain reaction results: a descriptive study. Canadian Medical Association Open Access Journal 11(5): E982–E987. 10.9778/cmajo.20230033 PMC10610021 37875313

[bibr11-18333583251370486] Regan AK Arah OA Sullivan SG (2022) Performance of diagnostic coding and laboratory testing results to measure COVID-19 during pregnancy and associations with pregnancy outcomes. Paediatric and Perinatal Epidemiology 36(4): 508–517. 35077581 10.1111/ppe.12863 PMC9303575

[bibr12-18333583251370486] Van Honacker E Van Vaerenbergh K Boel A , et al. (2021) Comparison of five SARS-CoV-2 rapid antigen detection tests in a hospital setting and performance of one antigen assay in routine practice: A useful tool to guide isolation precautions? Journal of Hospital Infection 114: 144–152. 33785377 10.1016/j.jhin.2021.03.021 PMC7999797

[bibr13-18333583251370486] World Health Organization (2020) International guidelines for certification and classification (coding) of covid-19 as cause of death based on ICD, Geneva, Switzerland.

[bibr14-18333583251370486] Wu G D’Souza AG Quan H , et al. (2022) Validity of ICD-10 codes for COVID-19 patients with hospital admissions or ED visits in Canada: A retrospective cohort study. BMJ Open 12(1): e057838. 10.1136/bmjopen-2021-057838 PMC8787827 35063962

