# Supplementary material for: Concordance between ICD-10-AM clinical coding and SARS-CoV-2 PCR testing for COVID-19 in Australian hospitals
Source: Health Inf Manag. 2025 Sep 12;55(3):458–63. doi: 10.1177/18333583251370486 (PMC13558799; doi:10.1177/18333583251370486)
Supplement: Supplementary file 1 [file sj-docx-1-him-10.1177_18333583251370486.docx]

**Supplementary files**

Table S1: COVID-19 related ICD-10-AM clinical codes (Eleventh Edition, NSW Australia, 2025) (IHACPA, 2022).

| **ICD-10 clinical codes** | **Diagnosis** | **Definition** |
| --- | --- | --- |
| U07.1 | Confirmed COVID-19 | Patient has laboratory-confirmed SARS-CoV-2 infection (PCR test positive). |
| U07.2 | Suspected or probable COVID-19 | Patient exhibits clinical symptoms indicative of COVID-19 without laboratory confirmation. Includes cases diagnosed clinically, supported by radiological evidence or positive rapid antigen test (RAT). |
| U07.3 | Personal history of COVID-19 | Patient has a documented history of confirmed COVID-19, currently resolved, and no longer infectious. |
| U07.4 | Post COVID-19 condition (Long COVID) | Patient experiences ongoing or new symptoms directly attributable to previous SARS-CoV-2 infection, persisting beyond the acute infection period. |
| U07.5 | Multisystem inflammatory syndrome associated with COVID‑19 (MIS-C/MIS-A) | Diagnosis of multisystem inflammatory syndrome secondary to confirmed or suspected COVID-19 infection. |
| U07.7 | Adverse effects related to COVID‑19 vaccination | Patient experiences clinically significant adverse reactions directly attributed to COVID-19 vaccination. |
| U06.0 | COVID-19 ruled out | Used when COVID-19 testing is conducted, and the results conclusively rule out SARS-CoV-2 infection. |
| B34.2 | Coronavirus infection, unspecified site | Assigned to classify asymptomatic infection, where documentation indicates a confirmed case of COVID-19 in an asymptomatic patient. B34.2 may be assigned as a principal diagnosis |
| Z03.81 | Observation for suspected exposure ruled out | Assigned when a person is observed or evaluated for possible exposure to a biological agent, such as a virus (e.g., SARS-CoV-2), but the diagnosis is ultimately ruled out. |

Table S2. Summary of selected published studies evaluating the accuracy of ICD-10 clinical code U07.1 compared to PCR-confirmed COVID-19 cases.

| Study | Year | Population | Sensitivity (%) | Specificity (%) | PPV (%) | NPV (%) |
| --- | --- | --- | --- | --- | --- | --- |
| [Kadri et al., 2020](https://jamanetwork.com/journals/jama/fullarticle/2774396) | 2020 | Hospitalised patients | 98.0 | 99.0 | 91.5 | 99.8 |
| [Lynch et al., 2021](https://www.tandfonline.com/doi/full/10.2147/CLEP.S335621?scroll=top&needAccess=true#d1e391) | 2021 | Inpatient | Not reported | Not reported | 93.8 | Not reported |
| [Bhatt et al., 2021](https://link.springer.com/article/10.1007/s11606-021-06936-w/tables/1) | 2021 | Hospitalised patients | 49.2 | 99.4 | 90.0 | 94.8 |
| [Moura et al., 2023](https://www.cmajopen.ca/content/11/5/E982?utm_source=TrendMD&utm_medium=cpc&utm_campaign=CMAJ_Open_TrendMD_1) | 2023 | Admitted Patients | 97.8 | 99.5 | Not reported | Not reported |
| [Regan et al., 2022](https://onlinelibrary.wiley.com/doi/full/10.1111/ppe.12863) | 2022 | Pregnant women | 60.7 | 92.8 | 36.2 | 97.2 |
| [Moura, Cristiano S., et al.](https://www.nature.com/articles/s41598-023-49501-7) | 2024 | Hospitalised patients | 93.6 | 99.8 | 98.6 | 98.8 |

Table S3: Results of mixed-effects logistic regression model, including adjusted odds ratios and 95% confidence intervals for all fixed-effect covariates.

| **Variable** | **OR** | **Lower CI** | **Upper CI** | **P-value** | **Model** |
| --- | --- | --- | --- | --- | --- |
| **ICD-10-AM U07.1 assigned** |  |  |  |  |  |
| Yes | 29.54 | 23.41 | 36.53 | <0.001 |  |
| No | Ref |  |  |  |  |
| **Age on admission (Years)** | 0.99 | 0.98 | 0.99 | <0.001 |  |
| Sex |  |  |  |  |  |
| Male | Ref |  |  |  |  |
| Female | 1.06 | 0.98 | 1.14 | 0.16 | Model 1 - Rapid PCR |
| **Socioeconomic grouping** |  |  |  |  |  |
| Low | 0.87 | 0.6 | 1.28 | 0.49 |  |
| Mid | 0.89 | 0.8 | 1 | 0.04 |  |
| High | Ref |  |  |  |  |
| **Year of admission** |  |  |  |  |  |
| **2022** | Ref |  |  |  |  |
| **2023/24** | 1.69 | 1.54 | 1.86 | <0.001 |  |
|  |  |  |  |  |  |
| **ICD-10-AM U07.1 assigned** |  |  |  |  |  |
| Yes | 17.73 | 12.91 | 23.21 | <0.001 |  |
| No | Ref |  |  |  |  |
| **Age on admission (Years)** | **0.99** | **0.98** | **0.99** | <0.001 |  |
| **Sex** |  |  |  |  |  |
| Male |  |  |  |  |  |
| Female | 1.06 | 0.99 | 1.15 | 0.11 | Model 2 - Conventional PCR |
| **Socioeconomic grouping** |  |  |  |  |  |
| Low | 0.82 | 0.51 | 1.31 | 0.4 |  |
| Mid | 0.81 | 0.72 | 0.9 | <0.001 |  |
| High | Ref |  |  |  |  |
| Year of admission |  |  |  |  |  |
| 2022 | Ref |  |  |  |  |
| 2023/24 | 1.9 | 1.75 | 2.07 | 0 |  |

Table S4 presented the Intra-Class Correlation Coefficients (ICCs) by Model and Clustering Level. The ICCs are reported for clustering at the hospital and patient levels for both Rapid PCR and Conventional PCR datasets. **Note:** Minimal clustering by hospital in the Rapid PCR group, indicating consistent coding and testing practices across facilities in this subgroup. A moderate degree of clustering by hospital in the Conventional PCR group, possibly reflecting variability in coding or testing protocols across sites. A substantial proportion of variability was attributable to individual patients in both models, indicating strong within-patient correlation, which justifies the inclusion of the patient-level random effect.

Table S4: Intra-Class Correlation Coefficients by Model at patient and hospital level.

| **Model** | **Level** | **ICC (%)** |
| --- | --- | --- |
| Model 1 - Rapid PCR | Hospital | 1.27 |
|  | Patient | 46.71 |
| Model 2 - Conventional PCR | Hospital | 20.69 |
|  | Patient | 51.93 |

Figure S1: ROC curves comparing the ability of ICD-10-AM clinical code U07.1 to predict SARS-CoV-2 PCR positivity, using mixed-effects logistic regression models for both rapid and conventional PCR testing datasets.
